# An Efficient Somatic Embryo Liquid Culture System for Potential Use in Large-Scale and Synchronic Production of *Anthurium andraeanum* Seedlings

**DOI:** 10.3389/fpls.2019.00029

**Published:** 2019-01-28

**Authors:** Guangdong Wang, Chuanying Xu, Shuo Yan, Bin Xu

**Affiliations:** ^1^College of Horticulture, Nanjing Agricultural University, Nanjing, China; ^2^College of Agro-grassland Science, Nanjing Agricultural University, Nanjing, China

**Keywords:** *Anthurium andraeanum*, somatic embryogenesis, liquid culture, PEM proliferation, SE development, SE germination

## Abstract

*Anthurium andraeanum* Lind. is the second most important tropical flower in the world flower market. Somatic embryogenesis and plant regeneration in *Anthurium* has been reported previously; however, a stable and effective method for its commercial use has not been available. In this study, an efficient somatic embryogenesis and liquid culture system for large-scale production of *A. andraeanum* seedlings was achieved. Building on previous research, this study investigated the main factors for proembryogenic mass (PEM) proliferation, somatic embryo (SE) development, and SE germination in *Anthurium*. The results showed that relatively low concentrations of plant growth regulators, mineral nutrition, and sucrose promoted PEM proliferation, SE formation, and germination in a liquid culture system. This system can be described as follows: PEMs were induced from leaf blade explants on Murashige & Skoog (MS) medium with half-strength MS macronutrients (1/2 MS) containing 2.0 mg L^-1^ 2,4-dichlorophenoxyacetic acid (2,4-D), 0.5 mg L^-1^ kinetin (KT), and 3% sucrose and were proliferated in ½ MS liquid medium containing 1.0 mg L^-1^ 2,4-D, 0.5 mg L^-1^ KT, and 3% sucrose. The highest proliferation coefficients were 5.11–5.16. PEMs were then transferred to MS medium with 1/8 MS macronutrients (1/8 MS) liquid medium containing 1% sucrose to develop into globular embryos and mature embryos. Finally, the mature embryos were placed on four layers of absorbent filter paper saturated with 1/8 MS liquid medium containing 1% sucrose for germination, and an average of 60 seedlings *per* gram SEs was obtained. This liquid culture system can be used in large-scale and synchronic production of *Anthurium* seedlings.

## Introduction

*Anthurium andraeanum* Lind. belongs to the Araceae family. It is famous for its showy spathe and beautiful foliage and has been the second most important tropical flower (next to orchid) in the world flower market (Dufour and Guerin, 2003; Teixeira da Silva et al., 2015). In 1956, growers in Holland first produced *Anthurium* for commercial purposes, mainly through seeding propagation or reproductive ramets (Kunisaki, 1980). Pierik et al. (1974) first reported successful establishment of a tissue culture system for *Anthurium* that was later adopted for commercial propagation in the industry.

Typically, *Anthurium* tissue cultures have been established by organogenesis from leaf, petiole, spathe, or axillary bud explants, and multiple shoots can be proliferated through a repeated callus-induction and regeneration process or axillary shoot proliferation (Pierik, 1976; Pierik et al., 1979; Geier, 1990; Martin et al., 2003; Puchooa, 2005; Teixeira da Silva et al., 2015). However, this method has several disadvantages, including low propagation efficiency, occasional somaclonal variation, and inhomogeneity (Kuehnle et al., 1992). Such limitations are among the key factors that cause the high market price of *Anthurium* seedlings. On the other hand, somatic embryogenesis is an effective micropropagation method that can overcome the shortcomings described above and has been successfully used for many kinds of economically valuable plants (Merkle et al., 1990; Duoue et al., 2006; Klimaszewska et al., 2015).

Somatic embryogenesis and plant regeneration in *Anthurium* was reported first by Kuehnle et al. (1992), and further studied from 1996 to 2016 (Matsumoto et al., 1996; Xin et al., 2006; Bautista et al., 2008; Pinheiro et al., 2013, 2014; Bhattacharya et al., 2016). In these studies, the combination of 1.0–4.0 mg L^-1^ of 2,4-dichlorophenoxyacetic acid (2,4-D) and 0.33–0.5 mg L^-1^ kinetin (KT) was found to be the key factor in proembryogenic mass (PEM) induction, and 0.5–1.0 mg L^-1^ N^6^-benzyladenine (6-BA) is also known to be important in somatic embryo (SE) development and plant regeneration. However, other factors influencing PEM proliferation and SE development were not compared systematically. Thus, a stable and effective system of somatic embryogenesis for the large-scale production of seedlings in *Anthurium* has not been available.

Clonal propagation through solid-medium tissue culture limits the mass production of *Anthurium* seedlings for commercial use because it requires highly skilled manual labor and lacks an automated production process. Somatic embryogenesis in liquid medium is a high efficient method that can solve this problem due to advantages such as rapid, homogeneous growth; thus, it can easily meet the requirements for automated production (Ruffoni and Savona, 2005). This technique has been used in micropropagation of conifer trees (Gupta and Timmis, 2005; Klimaszewska et al., 2015), *Medicago truncatula* (Duoue et al., 2006), sugarcane (Andibrisibe et al., 1994), and banana (Gómez Kosky et al., 2002), yet such a system had not been reported for *Anthurium*.

To establish a stable and effective somatic embryogenesis and propagation system for *Anthurium* using liquid culture, the main factors influencing embryogenic callus proliferation and SE development were compared in this study. By dividing the whole culture system into four steps—PEM induction, PEM proliferation, SE formation and SE germination—we optimized each step to establish an comlete liquid propagation system *via* somatic embryogenesis in *Anthurium*. Such a system could be useful for *Anthurium* seedling mass production for commercial use; additionally, this system could also provide a basic method for propagation using bioreactors for large-scale, synchronic production and genetic transformation of *Anthurium* seedlings in the future.

## Materials and Methods

### Pro-embryogenic Mass (PEM) Induction

The methods described by Kuehnle et al. (1992) and Xin et al. (2006) were used for PEM induction. Fully expanded leaf blades of aseptic seedlings of *A. andraeanum* ‘Amigo,’ ‘Valentino,’ and ‘Sonate’ were used as explants (‘Amigo’ was used for the factor experiments; ‘Valentino’ and ‘Sonate’ were used to test the optimized protocol on other cultivars. PEM induction was initiated by cutting leaves (not including the edges) into square pieces approximately 0.8 cm × 0.8 cm and culturing the pieces on solid Murashige & Skoog (MS) medium (Murashige and Skoog, 1962) with half strength MS macronutrients (1/2 MS) (1/2 MS = half-strength MS macronutrients) and containing 3% (w/v) sucrose, 2.0 mg L^-1^ 2,4-D, 0.5 mg L^-1^ KT, and 5.5% (w/v) agar, with the pH adjusted to 5.5. The leaf pieces were cultured for 45 days (d) in darkness at 25 ± 1°C. Dark brown non-embryogenic calluses were often induced earlier than PEMs, but did not develop further. By contrast, PEMs were induced at a later stage, but grew rapidly once induced and formed ball-like structures with nodulous appearance and fresh yellow color.

### Liquid Culture for PEM Proliferation

The granular PEMs, which formed from the edges of the sliced leaf blades, were then transferred into 150-ml flasks with 75 ml liquid medium containing the same nutrient and hormonal composition as the PEM induction medium except as otherwise stated. The liquid cultures were rotated at 100 rpm at 25 ± 1°C, under scattered light at 20 μmol m^-2^ s^-1^. Proliferated PEMs in good condition were selected for proliferation experiments.

To test the liquid medium composition for PEM proliferation, 2,4-D at 1.0, 2.0, 3.0, or 4.0 mg L^-1^ and sucrose at 2, 3, 4, 5, or 6% (w/v) were tested in all possible combinations by inoculation with 1.0 g PEM *per* 75 ml liquid medium. The basal medium was 1/2 MS containing 0.5 mg L^-1^ KT. The PEMs were transferred to the same liquid medium every 10 days during the culture period. The proliferation coefficients were recorded after 30 days culture and calculated as follows:

Proliferation coefficient=fresh weight of PEMs after 30 days of culture/fresh weight of inoculated PEMs.

The effect of initial inoculum amount was also studied with initial PEM inoculum at 1.0, 2.0, 3.0, or 4.0 g *per* 75 ml liquid medium with 3% sucrose and 2.0 mg L^-1^ 2,4-D. The basal medium was 1/2 MS containing 0.5 mg L^-1^ KT. The fresh weight and proliferation coefficient of PEMs was recorded by weighing every 5 days during a 60-days continuous culture period.

Each treatment was performed three times. The proliferation coefficients were calculated as follows:

Proliferation coefficient=fresh weight of PEMs after the specified time in culture (0−60days)/fresh weight of inoculated PEMs.

### Liquid Culture for Somatic Embryo (SE) Induction

The proliferated granular PEMs were used to induce SEs in liquid culture. MS medium containing different strengths of MS macronutrients (1/8, 1/4, 1/2, MS) and sucrose concentrations (0, 1, 2, 3%) were tested for their effect on SE induction in the absence of plant growth regulators (PGRs). The numbers of SEs of different sizes *per* 1.0 g PEMs were recorded after 15 and 30 days. The transfer interval was 10 days and the other culture conditions were the same as for PEM proliferation.

Liquid medium with different NH_4_NO_3_ levels relative to full-strength MS medium (i.e., 1/2, 1/4, 1/8, and 0 NH_4_NO_3_), 1% sucrose, and no PGRs was used for SE development on MS or 1/2 MS basal medium lacking NH_4_NO_3_. PEMs (1.0 g) were inoculated into 150-ml flasks containing 75 ml of liquid medium, and the SE number *per* gram PEMs was recorded after 30 days of culture. The transfer period was 10 days, and the other culture conditions were the same as for primary proliferation.

### Liquid and Raft Culture for SE Germination

First, different concentrations (0, 0.25, 0.5, 1.0 mg L^-1^) of PGRs (BA, KT, and gibberellic acid [GA_3_]) were compared for their effects on SE germination with 0.5 g SEs in 75 ml of liquid medium at a rotating speed of 100 rpm. Second, rotating liquid culture and raft culture were compared for their effect on SE germination with 1/8 MS basal medium and 1% sucrose, under 20 μmol s^-2^ s^-1^ light intensity. For raft culture, 0.5 g SEs were placed on four layers of absorbent filter paper saturated with liquid medium in a Petri dish, and were transferred to new medium every 15 days. The number of germinated SEs (i.e., those with root length longer than SE diameter) *per* gram of SEs was recorded after 30 days of culture.

### Statistical Analysis

All treatments were performed three times. Each treatment contained 10 Erlenmeyer flasks (liquid culture) or Petri dishes (raft culture). SPSS Base 8.0 software and Excel software for Windows were used for statistical analysis. Data were analyzed using the two-way ANOVA method.

## Results

We divided the whole somatic embryogenesis system into four steps: PEM induction, PEM proliferation, SE induction, and SE germination. The PEM induction system has been reported previously (Xin et al., 2006). In the present study, we optimized the remaining three steps in liquid culture.

### PEM Proliferation in Liquid Culture

**FIGURE 1:**
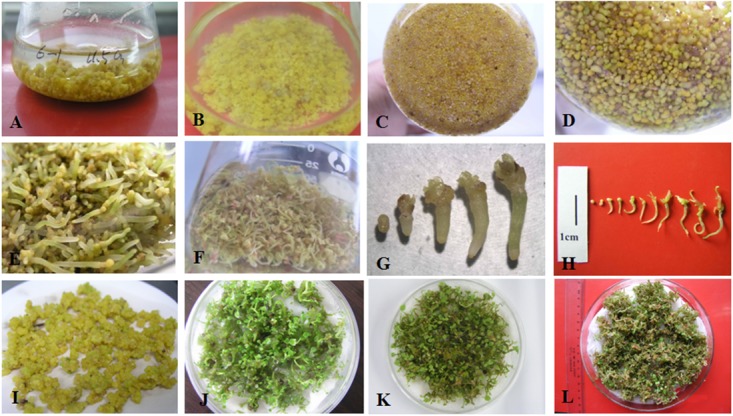
Somatic embryo proliferation, development, and germination of *Anthurium andraeanum.* **(A)** Embryogenic callus (PEMs) of ‘Amigo’ proliferating in liquid culture. **(B,C)** Embryogenic callus fragmented by tweezers during subculture. **(D)** Development of somatic embryos (SEs) after reducing the concentrations of PGRs, sucrose, and basal medium in liquid culture. Development and germination of SEs of ‘Amigo’ **(E)** and ‘Valentino’ **(F)** in liquid culture. Somatic embryogenesis of ‘Amigo’ **(G)** and ‘Valentino’ **(H)** grown in liquid culture. **(I)** Developed SEs of ‘Amigo’ in preparation for raft culture. Plantlets germinating in raft cultures of SEs of ‘Amigo’ **(J)**, ‘Valentino’ **(K)**, and ‘Sonate’ **(L)**.

To proliferate PEMs, we first tested 20 different combinations of 2,4-D and sucrose concentrations on their effects on PEM proliferation (Table 1). A pilot experiment showed that supplementation with 2,4-D and sucrose was necessary to maintain PEM status, yet with increasing concentrations of either 2,4-D or sucrose, the proliferation coefficient of PEMs decreased (Table 1). The highest proliferation coefficients (5.11–5.16) were achieved with 1.0 mg L^-1^ 2,4-D and 2–3% sucrose after 30 days of culture. In addition, the PEMs also became more nodulous and friable with lower concentrations of 2,4-D and sucrose (data not shown). In general, PEMs of the best quality (light yellow color and friable texture) were obtained with 1.0–2.0 mg L^-1^ 2,4-D and 3–4% sucrose (Figure 1A). Taking these observations together, we concluded that 1/2 MS supplemented with 1.0 mg L^-1^ 2,4-D, 3% sucrose, and 0.5 mg L^-1^ KT was the optimal medium for PEM proliferation in liquid culture.

**Table 1:** Effects of different concentrations of 2,4-D and sucrose on proliferation of *Anthurium* embryogenic callus (PEMs).

2,4-D concentration (mg L^-1^)	Sucrose (%)	Proliferation coefficient
1.0	2.0	5.11a
	3.0	5.16a
	4.0	4.32bc
	5.0	3.92c
	6.0	3.43de
2.0	2.0	4.58b
	3.0	2.98e
	4.0	2.28f
	5.0	2.23f
	6.0	2.16f
3.0	2.0	3.39de
	3.0	2.48f
	4.0	2.28f
	5.0	1.65g
	6.0	1.59g
4.0	2.0	3.46d
	3.0	1.66g
	4.0	1.66g
	5.0	1.64g
	6.0	1.63g


*The basal medium was 1/2 MS containing 0.5 mg L^-*1*^ KT. Data were measured after 30 days of culture. Means followed by the same letter were not significantly different at 0.05 level.*

**FIGURE 2:**
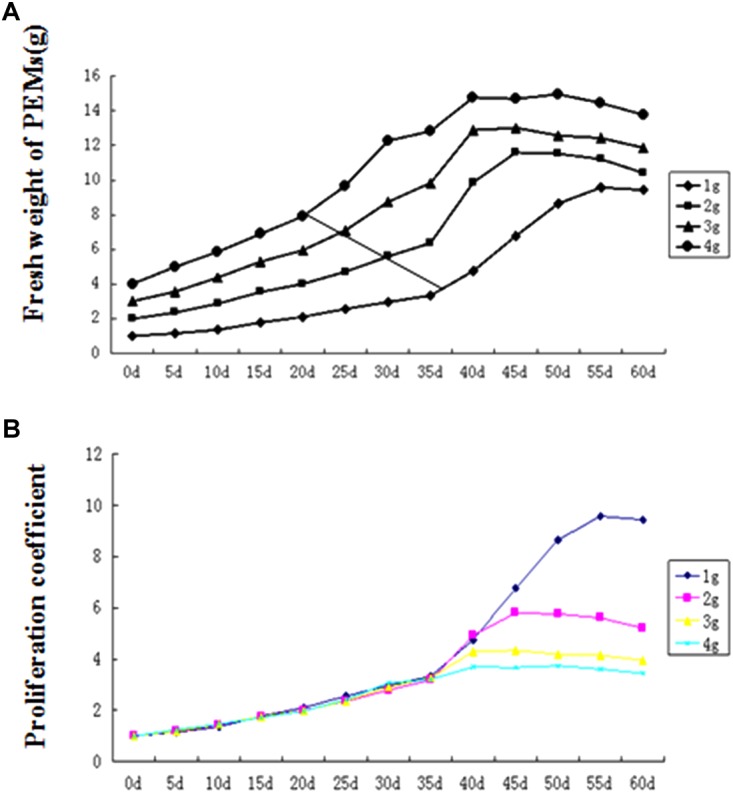
Proembryogenic mass (PEM) proliferation curves for different amounts of inoculum. **(A)** Proliferation rate curves (showing total fresh weight over time) for different inoculum amounts. **(B)** Proliferation coefficient curves (showing fresh weight of culture/fresh weight of inoculum) for different inoculum amounts. The diagonal line in **(A)** indicates the inflection point (between slow growth and logarithmic growth) in each curve.

In our pilot experiment, we also observed that under continuous culture without separation of the proliferated PEMs, the proliferation rate (i.e., the increase in fresh weight over time) followed an S-shaped curve. Therefore, we tested the effect of initial PEM inoculum amount on proliferation. As shown in Figure 2A, under continuous culture for 60 days, the proliferation rate curve can be divided into three stages: (1) initial slow growth, (2) logarithmic phase, and (3) stationary and decline phase. With higher amounts of initial inoculum, the inflection of the proliferation rate curve occurred earlier; in the logarithmic stage, the lower the amount of initial inoculum, the steeper the slope of the proliferation rate curve (corresponding to a higher proliferation coefficient; Figure 2B). As time in culture increased, the PEMs became more nodulous and scattered with some developing into SEs; the PEMs eventually turned brown and became necrotic at the stationary and decline stage. To avoid the occurrence of sporadic SE induction while maintaining a highly homogenous PEM culture, we concluded that the optimal inoculum amount for 75 ml of liquid medium was ∼1 g of PEM and the optimal transfer period was approximately 25 days (see Discussion for further explanation).

### Homogenous Somatic Embryo (SE) Induction in Liquid Culture

To induce somatic embryogenesis from PEMs, combinations of different strengths of MS basal medium and sucrose concentrations were tested. As shown in Table 2, reduced basal medium strength was critical for SE induction, and low sucrose concentration (1%) also improved SE induction. The best result was from the combination of 1/8 MS and 1% sucrose, which yielded an average of 126.7 SEs (≥1 mm diameter) out of 1.0 g inoculated PEMs by 15 days after inoculation. The induced SEs were also highly homogenous, i.e., nearly all induced SEs were at the globular stage (Figure 1D). On the other hand, with increased induction time, the number of SEs stayed the same after 30 days of culture, but the average size (≥2 mm) of the induced SEs increased (Table 2).

**Table 2:** Effects of basal medium strength and sucrose on somatic embryogenesis of *Anthurium* embryogenic callus (PEMs).

Basal medium strength	Sucrose (%)	15 days after inoculation			30 days after inoculation	
			
		SE number (diameter ≥1 mm)/g PEMs	SE number (diameter ≥2 mm)/g PEMs	SE number (diameter ≥3 mm)/g PEMs	SE number (diameter ≥2 mm)/g PEMs	SE number (diameter ≥3 mm)/g PEMs
MS	0	19.3f	0e	0c	18.66de	0c
	1	22.0f	0e	0c	2.66gh	0c
	2	12.7fg	0e	0c	0h	0c
	3	10.7fg	0e	0c	0h	0c
1/2 MS	0	5.3g	0e	0c	0h	0c
	1	11.3fg	0e	0c	8.66fgh	0c
	2	10.0fg	0e	0c	0h	0c
	3	0g	0e	0c	0h	0c
1/4 MS	0	5.3g	0e	0c	0h	0c
	1	53.3d	14.0c	0c	36c	0c
	2	34.0e	8.0d	0c	16ef	0c
	3	20.0f	0e	0c	0h	0c
1/8 MS	0	10.7fg	0e	0c	0h	0c
	1	126.7a	60.7a	17.3a	156a	25.34a
	2	88.0b	50.7b	12.0b	78b	11.34b
	3	71.3c	11.3cd	0c	24.66d	0c


*Data were measured after 30 days of culture. Means in the same column followed by the same letter were not significantly different at 0.05 level.*

In addition, we examined the effects of NH_4_NO_3_ on SE proliferation. The results showed that the concentration of NH_4_NO_3_ in the liquid medium had a very significant effect on SE development (Table 3). The number of SEs increased significantly with reduced NH_4_NO_3_ levels in either MS or 1/2 MS basal medium, but there was no overall difference between MS basal medium and 1/2 MS basal medium at the same NH_4_NO_3_ level according to ANOVA analysis.

**Table 3:** Effect of NH_4_NO_3_ on somatic embryogenesis of *Anthurium* embryogenic callus (PEMs).

Basal medium	Ratio of NH_4_NO_3_	Proliferation coefficient	SE number (≥1 mm)/gPEMs	SE number (≥2 mm)/gPEMs	SE number (≥4 mm)/gPEMs
MS	1/2	3.68e	10.0c	0e	0c
	1/4	4.46d	43.34b	5.34de	0c
	1/8	5.67a	50.0b	13.34c	0c
	0	5.35ab	75.34a	36.66a	16.0a
1/2 MS	1/2	3.66e	6.66c	0e	0c
	1/4	4.68cd	46.66b	5.34de	0c
	1/8	4.99bcd	50.0b	8.66cd	0c
	0	5.23abc	68.66a	30.66b	10.66b


*All media contained 1% sucrose and no PGRs. “Ratio of NH_*4*_NO_*3*_” indicates the NH_*4*_NO_*3*_ level relative to that found in full-strength MS medium. Data were measured after 30 days of culture. Means followed by the same letter were not significantly different at 0.05 level.*

There were green protuberances on the surface of the PEM when the NH_4_NO_3_ level was high. The SE and radicle developed better with reduced NH_4_NO_3_ levels, with a maximal SE number when cultured in MS or 1/2 MS basal medium without NH_4_NO_3_. However, the plumule pole was merged more significantly and the SEs were less homogeneous in the liquid medium with low NH_4_NO_3_ than in liquid medium containing 1/8 MS and 1% sucrose.

### SE Germination in Raft Culture

To test germination conditions, the homogenous SEs were germinated either in rotating liquid culture or in raft culture and with or without PGRs (BA, KT, GA_3_). Supplementation with BA, KT, or GA_3_ all suppressed SE germination, and raft culture was more effective for SE germination than rotating liquid culture (Table 4). The highest number of germinated seedlings from SE was achieved on raft culture without PGR supplementation; under these conditions, an average of 60 seedlings germinated from 1 g of SEs after 30 days of culture (Table 4). Moreover, in the raft cultures, radicles of the germinated SEs rapidly elongated with white root hairs, and shoots developed after radicle elongation. By contrast, in liquid culture, the radicles grew slower and with no root hairs, and secondary SEs often emerged at the pole of the plumule even in the absence of PGRs (Figure 1E–J). All the germinated SEs could grow into normal plantlets.

**Table 4:** Comparison of raft and rotating liquid culture and effects of plant growth regulators on germination of *Anthurium* somatic embryos.

Culture method	Plant growth regulator	Concentration (mg L^-1^)	No. of germinated seedlings *per* g of SEs
Raft culture	–	-	60.0a
	BA	0.25	13.3c
		0.5	12.0cde
		1.0	20.7b
	KT	0.25	12.7cd
		0.5	12.7cd
		1.0	12.7cd
	GA_3_	0.25	8.0cdefg
		0.5	4.7efg
		1.0	0.7g
Rotating liquid culture	–	-	15.3bc
	BA	0.25	5.3defg
		0.5	4.7efg
		1.0	3.3fg
	KT	0.25	5.3defg
		0.5	4.7efg
		1.0	9.3cdef
	GA_3_	0.25	2.0fg
		0.5	5.3defg
		1.0	4.7efg


*Data were measured after 30 days of culture. Means followed by the same letter were not significantly different at 0.05 level.*

### Additional Genotypes

This system development described above was performed using ‘Amigo.’ The optimized micropropagation system (see section “Conclusion”) was successfully applied to two other cultivars of *Anthurium*, ‘Valentino’ and ‘Sonate’ (Figure 1F,H,K,L).

## Discussion

Micropropagation *via* somatic embryogenesis is a more desirable method than *via* organogenesis from callus owing to high culture homogeneity and ease of using a low-cost liquid culture system (Merkle et al., 1990). Somatic embryogenesis and plant regeneration in solid culture has been reported in *Anthurium* (Kuehnle et al., 1992; Xin et al., 2006; Bautista et al., 2008; Pinheiro et al., 2013, 2014; Bhattacharya et al., 2016), but a complete liquid culture system including proliferation of PEMs, their inducible transition to homogenous SEs, and SE germination has not previously been reported for *A. andraeanum*.

### Key Factors in PEM Proliferation in Liquid Culture

Proembryogenic mass are in fact a solid cluster of SEs at an early development stage (von Arnold et al., 2002). The state of PEMs induced from leaf explants in the present study was similar to that of the embryogenic calli described by Kuehnle et al. (1992) and Bautista et al. (2008). In this study, PEMs rather than callus or developed mature SEs were used for proliferation because of the easier maintenance of the homogenous developmental state and the inducible transition from PEMs to SEs. It was reported that 2,4-D was very important for the induction of *Anthurium* SEs (Kuehnle et al., 1992; Hamidah et al., 1997b; Xin et al., 2006; Bautista et al., 2008). Regarding the somatic embryogenesis of *A. andraeanum* (Kuehnle et al., 1992) or *A. scherzerianum* (Hamidah et al., 1997b), relatively high concentrations of 2,4-D (18 μmol L^-1^) and carbohydrate (4% sucrose plus 2% glucose) can induce PEMs effectively. However, in our study, we found that the proliferation coefficient of PEMs cultured in liquid medium fell with increasing concentrations of 2,4-D and sucrose. The reason behind this might be that induction of PEM was related to stress that occurred under high osmotic pressure (e.g., by using a high concentration of sucrose; Stasolla and Yeung, 2003) and under high concentration of auxin, especially with 2,4-D, an auxin analog and a dicot herbicide when at high concentrations (Coca et al., 1994; Kitamiya et al., 2000; Fehér et al., 2001). Once PEMs were induced, their proliferation no longer required high concentration of 2,4-D. Furthermore, liquid culture allows rapid nutrient uptake by cells and speedy nutrient replacement at the cell surface *via* diffusion. Thus, lower nutrient levels are usually optimal during PEM proliferation compared to those typically used in solid media (Gupta and Timmis, 2005).

### Homogeneity of PEMs During Proliferation in Liquid Culture Condition

In plant cell suspension culture, the proliferation curves are typically S-shaped, as in the cases of *Angelica sinensis* (Oliv.) (Tsay and Huang, 1998), avocado (Witjaksono and Litz, 1999), and date palm (Fki et al., 2003). In this study, a long period of logarithmic growth was observed at the early stage of proliferation (Figure 2A). This might be because the proliferation materials were PEMs rather than suspension cells. In liquid culture, the outer layer of PEMs would be in full contact with culture medium, but the inner cells might be restricted from growth due to less growth stimulus from the culture medium and to physical restraint by the outer layers of PEMs. During the rotating liquid culture, the PEMs often fell apart into smaller, friable parts that adapted to the liquid culture condition (e.g., Figure 1B,C), thereby slowly reaching the flexion of proliferation.

On the other hand, the induction of SEs could sporadically occur during proliferation. Since our results showed that lower concentrations of PGRs or nutrients in the liquid medium could induce SEs, the appearance of SEs during the proliferation of PEMs in continuous culture might indicate that the PGRs and nutrients were gradually depleted during culture. It was also found in other plant species that lower concentrations of PGRs could induce SEs (Chol et al., 1999; Stasolla and Yeung, 2003). Therefore, we suggest that the inoculum amount and transfer period should be well-controlled, even at the expense of reducing the proliferation coefficient, to maintain highly homogenous PEMs because homogeneity could be one of the most important factors for commercial micropropagation. In the present study, the optimal inoculum amount for 75 ml liquid medium was approximately 1.0 g, and the optimal transfer period was approximately 25 days.

### Low Nutrient Concentration Was Critical for the Induction of SE in *Anthurium*

Previously, different combinations of 6-BA or KT and their effects on SE induction, development, and germination have been reported in *A. andraeanum* (Kuehnle et al., 1992; Xin et al., 2006; Bautista et al., 2008) and *A. scherzerianum* (Hamidah et al., 1997b), yet our experimental results showed that lower levels of nutrients and sucrose, rather than PGRs, were critical for homogenous SE induction. We have also tested the effects of abscisic acid (ABA) on the development, maturation, and germination of SEs, but no significant differences were ever detected (data not shown), which was consistent with a report on *A. scherzerianum* (Hamidah et al., 1997a).

It was reported that a low level of NH_4_NO_3_ (200 mg L^-1^) was beneficial for inducing regeneration in all genotypes of *A. scherzerianum* (Geier, 1986). Our experiment also showed that gradual reduction of NH_4_NO_3_ in MS and 1/2 MS basal medium promoted SE formation, but the effect was not as strong as that obtained by reducing all of the MS macronutrients, e.g., to 1/8 MS (Tables 2, 3). The fact that lower levels of nutrients promoted SE induction was probably because SE formation was responsive to nutrient deficiency, similar to what had been found with cotton (*Gossypium hirsutum*) (Kumria et al., 2003).

### Depletion of PGRs Promoted SE Germination Under Raft Culture Conditions

Both 6-BA and KT at low levels are common PGRs used in SE germination in some plants, such as rice (*Oryza sativa*), *Camellia nitidissima*, and *Asparagus breslerianus* (Rueb et al., 1994; Lü et al., 2013; Mousavizadeh et al., 2017), and these PGRs have also been used for SE germination in *A. andraeanum* (Kuehnle et al., 1992; Xin et al., 2006; Bautista et al., 2008) and *A. scherzerianum* (Hamidah et al., 1997b). GA_3_ is another PGR known for promoting SE germination in plants such as *Eleutherococcus senticosus* and *Dianthus* (Chol et al., 1999; Pareek and Kothari, 2003). However, in our experiment, supplementation with 6-BA, KT, or GA_3_ at 0.25–1.0 mg L^-1^ inhibited the germination of SEs. Instead, KT and GA_3_ caused the formation of secondary embryos, while BA induced the formation of non-embryogenic callus (data not shown). This discrepancy between our study and previous studies could be due to the culture condition of SEs (liquid vs. solid-medium culture). In solid culture media (e.g., those solidified with agar or gellan gum), the uptake of nutrients by SEs was relatively slow, which could generate concentration gradients for each nutrient in the zone of the gel next to the cells (Gupta and Timmis, 2005). Liquid medium allowed the accumulation of nutrients and PGRs in the body of cultures during proliferation, at an earlier stage of development. Additional supplementation with extra PGRs could suppress germination of SEs. Compared to the submerged rotating liquid culture, the raft culture system was superior for SE germination, which could be due to the optimum oxygen concentration, low shear force, and normal gravity in the raft culture. In addition, raft culture provided lower water content conditions. It has been reported that desiccation treatment improved SE conversion and germination in alfalfa (*Medicago sativa*) and conifers (Lai and McKersie, 1994; von Arnold et al., 2002; Klimaszewska et al., 2015; Zhou et al., 2017). Our results were consistent with these previous reports.

## Conclusion

**FIGURE 3:**
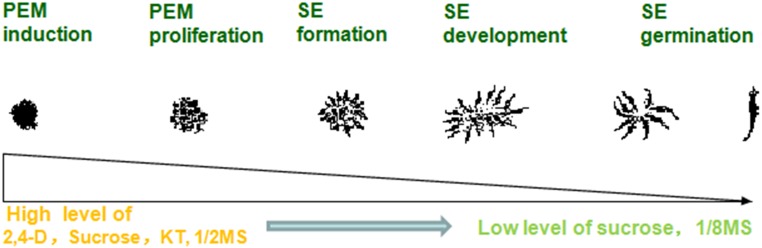
Process of somatic embryogenesis from PEM induction to SE germination in *Anthurium* liquid culture system.

A highly stable micropropagation system was established *via* somatic embryogenesis in *A. andraeanum* ‘Amigo.’ In the PEM induction stage, higher concentrations of 2,4-D, KT, and sucrose are necessary. However, lower concentrations of 2,4-D, mineral nutrition, and sucrose promoted PEM proliferation and SE development. Absorbent filter paper with decreased nutrition and hormone-free medium was suitable for SE germination (Figure 3).

This micropropagation system has been successfully applied to two other cultivars of *Anthurium*: ‘Valentino’ and ‘Sonate.’ Once PEMs were induced, PEM proliferation and SE development was very effective for *in vitro* seedling propagation of these two cultivars. However, specific optimizations might still be necessary for some other varieties.

**FIGURE 4:**
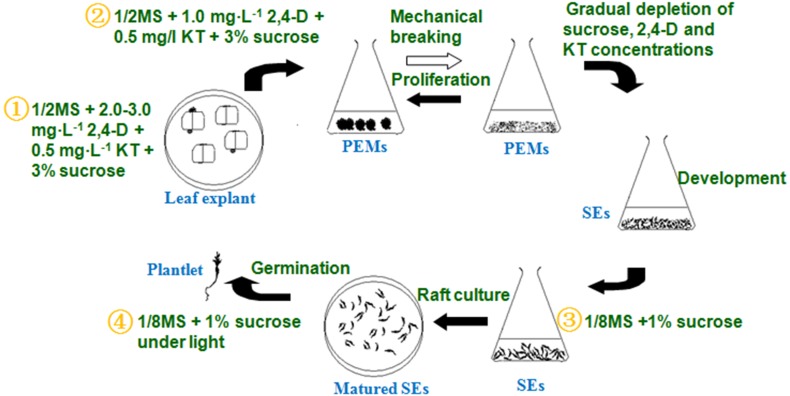
Detailed liquid micropropagation procedure for *A. andraeanum* ‘Amigo’ *via* SEs.

The optimized somatic embryogenesis procedure can be summarized as follows (Figure 4):

1. PEM induction (∼45 days): PEMs were induced from leaf blade explants on 1/2 MS solid medium containing 2–3 mg L^-1^ 2,4-D, 0.5 mg L^-1^ KT, 3% sucrose, and 5.5% agar.
2. PEM proliferation (∼30–60 days): PEMs were proliferated in 1/2 MS liquid medium containing 1.0 mg⋅L^-1^ 2,4-D, 0.5 mg⋅L^-1^ KT, and 3% sucrose. Inoculum was 1.0 g PEMs *per* 75 ml liquid culture, transferred every 25 days.
3. SE induction (∼30 days): PEM fragments were transferred to 1/8 MS liquid medium containing 1% sucrose to develop into globular embryos.
4. SE germination (∼30–60 days): Mature embryos were added to four layers of absorbent filter paper saturated with 1/8 MS liquid medium containing 1% sucrose for germination.

This liquid culture system provided a basic technique for large-scale, synchronic production of SEs and seedlings in *Anthurium*, which could have high commercial benefit. It also provided a basic technique for *in vitro* breeding, such as through genetic transformation or embryonic mutant induction.

Although the germinated seedlings from this system were identical in organogenesis and phenotype (data not shown), somaclonal variation is a problem in some species (von Arnold et al., 2002). Further studies are needed to determine whether somaclonal variation occurs in *Anthurium* seedlings developed by using this method.

## Author Contributions

GW designed the experiment and wrote the manuscript. CX, SY, BX, and GW performed the experiment and data analysis.

## Conflict of Interest Statement

The authors declare that the research was conducted in the absence of any commercial or financial relationships that could be construed as a potential conflict of interest.
